# Inhalation of virus-loaded droplets as a clinically plausible pathway to deep lung infection

**DOI:** 10.3389/fphys.2023.1073165

**Published:** 2023-01-19

**Authors:** Aranyak Chakravarty, Mahesh V. Panchagnula, Neelesh A. Patankar

**Affiliations:** ^1^ School of Nuclear Studies and Application, Jadavpur University, Kolkata, India; ^2^ Department of Applied Mechanics, Indian Institute of Technology Madras, Chennai, India; ^3^ Department of Mechanical Engineering, Northwestern University, Evanston, IL, United States

**Keywords:** SARS-CoV-2, influenza, mucociliary clearance, weibel model, infection kinetics, pneumonia onset time, vaccination efficacy, aerosolization

## Abstract

Respiratory viruses, such as SARS-CoV-2, preliminarily infect the nasopharyngeal mucosa. The mechanism of infection spread from the nasopharynx to the deep lung–which may cause a severe infection—is, however, still unclear. We propose a clinically plausible mechanism of infection spread to the deep lung through droplets, present in the nasopharynx, inhaled and transported into the lower respiratory tract. A coupled mathematical model of droplet, virus transport and virus infection kinetics is exercised to demonstrate clinically observed times to deep lung infection. The model predicts, in agreement with clinical observations, that severe infection can develop in the deep lung within 2.5–7 days of initial symptom onset. Results indicate that while fluid dynamics plays an important role in transporting the droplets, infection kinetics and immune responses determine infection growth and resolution. Immune responses, particularly antibodies and T-lymphocytes, are observed to be critically important for preventing infection severity. This reinforces the role of vaccination in preventing severe infection. Managing aerosolization of infected nasopharyngeal mucosa is additionally suggested as a strategy for minimizing infection spread and severity.

## 1 Introduction

Respiratory viruses, like the severe acute respiratory syndrome coronavirus 2 (SARS-CoV-2) which causes the disease COVID-19, are transmitted mainly through virus-laden droplets (Harrison et al., 2020; Wiersinga et al., 2020). The droplets originate from an infected individual, which in turn can be expelled into the environment by various mechanisms (talking, sneezing, coughing, and even normal exhalation) (Abkarian et al., 2020; Abkarian and Stone, 2020). The expelled droplets may subsequently be inhaled by other individuals; often after face-to-face exposure. Once inhaled, the droplets are subjected to advective-diffusive transport in the respiratory tract through the breathing dynamics and may eventually deposit in the respiratory mucosa in different regions of the respiratory tract (Mittal et al., 2020). SARS-CoV-2 has been observed to initially deposit and replicate in the upper respiratory tract (URT), particularly the nasopharynx (Grant et al., 2021), although there remains a possibility of the deposition taking place directly in the lower respiratory tract (LRT) depending on the inhaled droplet size and flow conditions (Madas et al., 2020; Chakravarty et al., 2022). It is, however, unlikely that the initial infection will be seeded in the LRT. The fraction of droplets that get deposited in the LRT is much less (∼17%) as compared to the URT (∼65%) (Sznitman, 2013; Madas et al., 2020). The LRT also has a much larger surface area as compared to the URT (Menache et al., 1997; Fröhlich et al., 2016). In addition, the mucus clearance mechanism continuously removes any viral deposition from the LRT towards the URT. Several physiological mechanisms also play an active role in neutralizing the viruses in the LRT. Lastly, the receptor cells required for the deposited viruses to replicate are less abundant in the LRT, as compared to URT (Wiersinga et al., 2020; Jackson et al., 2022). As such, a much higher viral load ( >4 times the URT viral load) is required to initiate infection in the LRT than that in the URT. It is unlikely that such a large viral load can be inhaled from environmental exposure and hence, it is more probable that the initial infection will be seeded in the URT.

Once the infection is seeded in the nasopharynx, the virus releases its ribonucleic acid (RNA) and utilizes the host cell machinery to create and release hundreds of new virions resulting in rapid progression of infection. Severity of infection and associated health complications (e.g., pneumonia, acute respiratory distress syndrome (ARDS)), however, depend on whether the virus can gain access to the distal regions of the thorax and the lung, particularly the alveoli. Autopsy studies on patients with COVID-19 who had developed respiratory failure has confirmed the existence of SARS-CoV-2 in the alveolar epithelial cells as well as in alveolar macrophages (Hou et al., 2020; Grant et al., 2021). The mechanism by which virions are transported from the URT to the alveolar region is, however, still an open question. Further multiplication in the LRT and gastrointestinal mucosa also results in mild viremia suggesting a systemic nature of the disease (Chen et al., 2020).

One hypothesis for the migration of the infection from the nasopharynx to the deep lungs (alveolar region) could be through virus diffusion in respiratory mucus if mucociliary clearance is impaired (Karamaoun et al., 2018). However, the time required for the viruses to diffuse (while replicating) across the entire length of a lung is too long to be probable (Chen et al., 2022). The total length of a typical healthy human lung is ∼0.28 m (Weibel, 1963), while diffusion coefficient of SARS-CoV-2 is typically $∼O\left(7\times 10^{-13}{\text{ m}}^{2}/s\right)$ [considering virus size to be 60 nm (Wiersinga et al., 2020)], resulting in a time-scale of more than 3,000 years (time scale ∼length^2^/diffusivity)! Hematogenous viremia is another possible route for the virus to reach the distal lungs. However, it only explains a small fraction of the fatalities associated with SARS-CoV-2 (Hagman et al., 2020; Tan et al., 2020). Similarly, aspiration is a possible mechanism by which the nasopharyngeal fluid can reach the alveolar region (Basu, 2021; Basu et al., 2022). Another possibility is through inhalation of infected droplets, present in the nasopharynx, deeper into the LRT. These may be the droplets inhaled from the enivronment (which have not deposited in the URT), or those formed due to aerosolization of infected nasopharyngeal mucosa (ANM) (Edwards et al., 2021; Darquenne et al., 2022). Hydrodynamic interaction between breathed air and the infected nasopharyngeal mucosa may cause the latter to aerosolize in certain conditions creating virus-laden aerosols/droplets (Moriarty and Grotberg, 1999). While most of these aerosols/droplets are exhaled out, some may get retained within the nasopharynx allowing further inhalation into the LRT where they may again deposit releasing the viruses and thereby, spread the infection to different regions of the LRT, including the deep lung. The plausability of infection spreading to the deep lung through inhalation of such pharyngeal droplets is investigated in this work. Droplets/aerosols of different sizes are considered. However, all sizes will be referred to as droplets in this work to avoid confusion.

In order to explore the plausibility of this mechanism, one needs a mathematical model which includes droplet (in airways) and virus (in mucus) transport within the LRT, along with virus infection kinetics. Extensive independent studies have been carried out over the years on all these aspects (Baccam et al., 2006; Smith et al., 2008; Hofmann, 2011; Mauroy et al., 2011; Handel et al., 2018; Karamaoun et al., 2018; Jensen et al., 2019). A few studies have investigated the coupled nature of droplet and mucus transport (Chakravarty et al., 2022) as well as virus transport and infection kinetics (Quirouette et al., 2020). However, the nature of the phenomena requires simultaneous consideration of all the processes necessitating a fully coupled mathematical model taking into account the individual processes. Such a model is being reported for the first time. This model is used within the framework of a Weibel-type model of the complete human LRT, with appropriate modifications (Weibel, 1963; Chakravarty et al., 2022). Aerosolisation of nasopharyngeal mucosa is not explicitly considered in the present study. Instead, it is implicitly assumed that aerosolization of nasopharyngeal mucosa leads to formation of infected droplets which, along with the residual inhaled droplets, remain present in the pharyngeal region to be inhaled into the LRT.

The primary goal is to use this mathematical model to understand the onset of SARS-CoV-2 infection in different regions of the LRT, particularly the deep lung, through inhalation of carrier droplets present in the pharynx, and the subsequent infection progression in the presence of mucociliary clearance and virus diffusion. Different situations are analysed through pertinent dimensionless parameters with respect to their impacts on infection severity and infection resolution. The role of immune responses and vaccination is particularly highlighted. In addition, the time required post symptoms onset for development of pneumonia from a severe infection is also estimated from the results obtained.

## 2 Methods

The mathematical model is developed considering a one-dimensional *trumpet* model, with appropriate modifications, to approximate the dichotomous structure of the LRT in a human being (see Supplementary Figure S1 for more details). The following sections discuss the dimensionless equations governing droplet and virus transport as well as virus kinetics.

### 2.1 Droplet transport model

The one-dimensional transport equation for droplets in the modelled LRT can be represented in a dimensionless manner as (Chakravarty et al., 2022)

$$\begin{array}{cc} Pe_{d}St_{a}{\left(2\alpha \beta \right)}^{N}\frac{\partial \left(\phi_{d}\right)}{\partial \tau }= & \frac{\partial }{\partial N}\left[\left({\left(\frac{2\beta }{\alpha }\right)}^{N}{\left(\frac{1-\alpha }{\alpha \text{ln}\alpha }\right)}^{2}\frac{\partial \phi_{d}}{\partial N}\right)\right.\\ & \left.+\left(Pe_{d}q\left(\tau \right)\left(\frac{1-\alpha }{\alpha \text{ ln}\alpha }\right)\phi_{d}\right)\right]-L_{D}^{\prime }\phi_{d}, \end{array}$$ (1)

where *τ*, *ϕ*_*d*_, *Pe*_*d*_ and *St*_*a*_ represent dimensionless time, dimensionless droplet concentration in the airways, droplet Peclet number and airway Strouhal number, respectively. *N* is the generation number representing spatial position within the LRT, while *α* and *β* are length-change and area-change factors assumed while simplifying the geometry (see Supplementary Material for more details). The term on the left hand side of Eq. 1 takes into account temporal change in droplet concentration. The first and second terms on the right hand side of Eq. 1 takes into account droplet diffusion and advective transport, respectively, while the last term takes into account droplet deposition. Detailed derivation of Eq. 1 is provided in the Supplementary Material.

While formulating this model, it is assumed that the droplets are monodispersed, do not coagulate, and do not affect airflow in the airways. The only source of droplets is assumed to be in the pharynx which opens into the trachea (*N* = 0). These droplets may be a combination of the droplets inhaled during respiration (which have not deposited in the URT) or droplets that are formed through ANM. No additional aerosolization of the mucosa or droplet source are considered. The inhaled droplets are either deposited or washed out of the LRT. Major droplet deposition mechanisms *viz.* diffusion, sedimentation and impaction have been taken into account while calculating droplet deposition in the LRT (see *S1 Text* of Chakravarty et al. (2022) for details). The quantity $L_{D}^{\prime }\phi_{d}$ quantifies dimensionless deposition of the droplets.

The following scaling parameters are used to obtain Eq. 1

$$\tau =\frac{t}{T_{b}},\phi_{d}=\frac{c_{d}}{c_{d,0}},T_{a}=\frac{L_{0}A_{0}}{\left|Q_{\max}\right|},St_{a}=\frac{T_{a}}{T_{b}},Pe_{d}=\frac{|Q_{\max}|L_{0}}{A_{0}D_{d}}$$ (2)

where *T*_*b*_ and *T*_*a*_ represents the breathing time period and the airflow timescale, respectively. *c*_*d*_ is the dimensional droplet concentration with *c*_*d*,0_ representing the initial *c*_*d*_ at *N* = 0. *D*_*d*_ represents droplet diffusivity in air $\left(D_{d}=\left(k_{B}TC_{S}\right)/\left(3\pi \mu_{a}d_{d}\right)\right)$ and is calculated using the Stokes-Einstein relation (Chakravarty et al., 2019). *k*_*B*_, *T*, *C*_*S*_, *μ*_*a*_, and *d*_*a*_ are the Boltzmann constant, air temperature, Cunningham slip correction factor, air viscosity, and droplet diameter, respectively. It is important to note that *Pe*_*d*_ refers to the droplet Peclet number at *N* = 0 only. Thus, even if *Pe*_*d*_ becomes extremely large, the local droplet Peclet numbers in the deeper generations (higher *N*) can remain small. *Q* represents the volume flow rate of air during breathing and is modelled such that *Q* = *Q*_0_ *q*(*τ*), *q*(*τ*) being a sinusoidal function accounting for airflow variation during breathing. *L*_0_ and *A*_0_ are the airway length and cross-sectional area at *N* = 0, respectively.

### 2.2 Virus transport model

The only source of viruses in the LRT is the carrier droplets that deposit in the mucosa of the LRT. The deposited viruses diffuse in the mucosa and are also subjected to mucociliary advective transport. The corresponding transport equation of the deposited viruses in the mucosa is formulated considering these transport mechanisms in addition to kinetics of the virus infection (see Supplementary Material for more details). The one-dimensional virus transport equation, thus formulated, is represented in its dimensionless form as

$$\begin{array}{cc} Pe_{v}{\left(2\alpha \zeta \sqrt{\beta }\right)}^{N}St_{m}\frac{\partial \phi_{v}}{\partial \tau }= & \frac{\partial }{\partial N}\left[\left({\left(\frac{2\zeta \sqrt{\beta }}{\alpha }\right)}^{N}{\left(\frac{1-\alpha }{\alpha \text{ ln}\left(\alpha \right)}\right)}^{2}\frac{\partial \phi_{v}}{\partial N}\right)\right.\\ & \left.-\left(Pe_{v}{\left(2\varepsilon \zeta \sqrt{\beta }\right)}^{N}\phi_{v}\right)\right]+\left(L_{D}^{\prime }\frac{D_{d}}{D_{v}}\phi_{d}\right)\\ & +{\left(2\alpha \zeta \sqrt{\beta }\right)}^{N}\left(p_{0}I-c_{l}\phi_{v}\right), \end{array}$$ (3)

where *ϕ*_*v*_, *Pe*_*v*_ and *St*_*m*_ denotes the dimensionless virus concentration in mucus, virus Peclet number and mucus Strouhal number, respectively. The quantity $\left(L_{D}^{\prime }\frac{D_{d}}{D_{v}}\phi_{d}\right)$ takes into account the deposition of virus in mucus through the virus-laden droplets, while the last term takes into account virus infection kinetics using a modified target-cell limited model (Baccam et al., 2006; Quirouette et al., 2020). The first and second terms on the right hand side of Eq. 1 take into account the diffusive and advective transport of viruses in mucus, respectively. Temporal change in virus concentration is taken into account by the term on the left hand side of Eq. 3. It is assumed that the rate at which the uninfected target cells at any spatial location are infected is dependent on the infection rate and the local virus concentration (see Eq. 4). Once infected, the target cells remain in an eclipse phase for a certain time-span before they become infectious (Eq. 5). The infectious cells produce new virus at a specific rate for a certain duration before undergoing apoptosis (Eq. 6).

$$\frac{\partial T}{\partial \tau }=-I_{r}T\phi_{v}$$ (4)

$$\frac{\partial E}{\partial \tau }=\left(I_{r}T\phi_{v}\right)-\left(\frac{1}{\tau_{E}}E\right)$$ (5)

$$\frac{\partial I}{\partial \tau }=\left(\frac{1}{\tau_{E}}E\right)-\left(\frac{1}{\tau_{I}}I\right)$$ (6)

The fraction of uninfected target cells, infected cells in the eclipse phase and infectious cells are represented as *T*, *E* and *I*, respectively. *I*_*r*_ represents the dimensionless infection rate, while *τ*_*E*_ and *τ*_*I*_ are the dimensionless time-period of the eclipse phase and infectious phase, respectively. *p*_0_ is the dimensionless replication rate of virus from the infectious cells and *c*_*l*_ is the dimensionless virus clearance rate due to various non-specific clearance mechanisms (Baccam et al., 2006; Quirouette et al., 2020). The following scaling parameters are used to obtain Eqs. 3–6 (see Supplementary Material for detailed derivation)

$$\begin{array}{cc} \tau =\frac{t}{T_{b}}, & \phi_{v}=\frac{c_{v}}{c_{v,0}},St_{m}=\frac{T_{m}}{T_{b}},Pe_{v}=\frac{|V_{m,0}|L_{0}}{D_{v}},p_{0}=\frac{L_{0}^{2}}{D_{v}}\frac{p}{c_{v,0}},\\ & c_{l}=\frac{L_{0}^{2}}{D_{v}}c,I_{r}=\beta c_{v,0}T_{b},\tau_{E}=\frac{T_{E}}{T_{b}},\tau_{I}=\frac{T_{I}}{T_{b}} \end{array}$$ (7)

where *c*_*v*_ is the dimensional virus concentration and *c*_*v*,0_ is the initial *c*_*v*_ at *N* = 0 due to droplet deposition. *V*_*m*,0_ is the mucociliary advective velocity at *N* = 0. *T*_*m*_ and *D*_*v*_ are the time-scale for mucociliary advection and virus diffusivity, respectively. *p*, *c*_*l*_ and *β* represents the dimensional virus replication rate, virus clearance rate and infection rate, respectively. *T*_*E*_ and *T*_*I*_ are the time-scales for the eclipse phase and the infectious phase, respectively. *c*_*v*,0_, *T*_*m*_ and *D*_*v*_ are determined as

$$c_{v,0}=\phi_{l}c_{d,0}\frac{A_{0}}{A_{m,0}},T_{m}=\frac{L_{0}}{\left|V_{m,0}\right|},D_{v}=\frac{k_{B}T}{3\pi \mu_{m}d_{v}}$$ (8)

### 2.3 Immune response model

The impact of a human body’s immune response to viral infections in the LRT is taken into account through a simplified immune response model, following Quirouette et al. Quirouette et al. (2020), which considers innate immune response due to interferons, humoral immune response through antibodies and cellular immune response due to cytotoxic *T*-lymphocytes. The mathematical models used for these immune responses are discussed in the following sections.

#### 2.3.1 Interferon response

Interferons are assumed to attenuate virus replication from the infectious cells. The virus replication rate $\left(p_{0}^{\prime }\right)$ in presence of interferons is determined as

$$p_{0}^{\prime }=\left(1-\frac{F}{F+f}\right)p_{0}$$ (9)

where *p*_0_ is the virus replication rate in absence of interferons (see Eq. 3) and *f* is the fraction of interferons required to halve the virus replication rate. *F* is the fractional amount of interferons present in the body (relative to the maximum amount intereferons that may be present) and it varies with time as

$$F=\frac{2}{e^{-\lambda_{g,i}\left(\tau -\tau_{p,i}\right)}+e^{\lambda_{d,i}\left(\tau -\tau_{p,i}\right)}}$$ (10)

where *λ*_*g*,*i*_ and *λ*_*d*,*i*_ are the dimensionless growth rate and decay rate of interferons, respectively. *τ*_*p*,*i*_ is the dimensionless time at which amount of interferon reaches its maximum.

#### 2.3.2 Antibody response

The presence of antibodies increases the probability of neutralising the viruses present in the body. The clearance rate of viruses (*c*_*l*_ in Eq. 3), thus, gets enhanced in the presence of antibodies as

$$c_{l}^{\prime }=c_{l}+k_{A}^{\prime }Ab$$ (11)

where $c_{l}^{\prime }$ is the enhanced clearance rate in presence of antibodies and $k_{A}^{\prime }$ is the dimensionless binding affinity of antibodies to the viruses. *Ab* is the fraction of antibodies which varies with time as

$$Ab=\frac{1}{1+\left(\frac{1}{Ab_{0}}-1\right)e^{-\lambda_{g,a}\tau }}$$ (12)

where *λ*_*g*,*a*_ is the dimensionless growth rate of antibodies and *Ab*_0_ is the initial fraction of antibodies present.

#### 2.3.3 *T* − lymphocyte response

The *T* − lymphocytes are cytotoxic in nature and act by directly attacking the infected cells in their eclipse and infectious phases. Eqs 5, 6 are, thus, modified in presence of *T* − lymphocytes as

$$\frac{\partial E}{\partial \tau }=\left(I_{r}T\phi_{v}\right)-\left(\frac{1}{\tau_{E}}E\right)-\left(k_{c}^{\prime }T_{l}\right)E$$ (13)

$$\frac{\partial I}{\partial \tau }=\left(\frac{1}{\tau_{E}}E\right)-\left(\frac{1}{\tau_{I}}I\right)-\left(k_{c}^{\prime }T_{l}\right)I$$ (14)

where $k_{C}^{\prime }$ represents the dimensionless rate at which the infected cells are neutralised by the *T* − lymphocytes. *T*_*l*_ is the fractional amount of *T* − lymphocytes present at any time and is determined as

$$T_{l}=\frac{2}{e^{-\lambda_{g,t}\left(\tau -\tau_{p,t}\right)}+e^{\lambda_{d,t}\left(\tau -\tau_{p,t}\right)}}$$ (15)

where *λ*_*g*,*t*_ and *λ*_*d*,*t*_ are the dimensionless growth rate and decay rate of *T* − lymphocytes, respectively. *τ*_*p*,*t*_ is the dimensionless time at which amount of *T* − lymphocytes reaches its maximum.

### 2.4 Initial and boundary conditions

The LRT is assumed to be initially devoid of droplets and viruses, i.e., *ϕ*_*d*_|_*τ*=0_ = *ϕ*_*v*_|_*τ*=0_ = 0. Fractions of target cell and infected cells are also assumed to be initially zero (*T*|_*τ*=0_ = *E*|_*τ*=0_ = *I*|_*τ*=0_ = 0). The trachea (*N* = 0) is assumed to be exposed to virus-laden droplets, presumably a combination of inhaled droplets (which have not deposited in the URT) or those formed due to ANM, for a specific inhalation period (*τ*_*inh*_). The droplets are transported deeper into the LRT, along with the airflow, during inhalation (Eq. 16) and washed out during exhalation (Eq. 17). In contrast, the viruses are always assumed to be washed out of the trachea (*N* = 0) along with mucus, irrespective of inhalation/exhalation (Eq. 18), due to the nature of mucociliary transport. At the distal end of the lungs (*N* = 23), the total advection-diffusion flux of both droplets and viruses is assumed to be zero (Eq. 19). Mathematically, these conditions are expressed as follows

$$\begin{array}{cc} {\left.\phi_{d}\right|}_{N=0} & =1,\tau \leq \tau_{\mathrm{inh}},\\ & =0,\tau >\tau_{\mathrm{inh}}, \end{array}$$ (16)

$${\left.\frac{\partial F_{d}}{\partial N}\right|}_{N=0}=0,\tau >0,$$ (17)

$${\left.\frac{\partial F_{v}}{\partial N}\right|}_{N=0}=0,\tau >0,$$ (18)

$$F_{d}{|}_{N=23}=F_{v}{|}_{N=23}=0,\tau >0,$$ (19)

where *F*_*d*_ and *F*_*v*_ are the total advection-diffusion flux in the droplet transport (Eq. 1) and virus transport equation (Eq. 3), respectively.

### 2.5 Model validation and parameter estimation

The implemented mathematical model is validated with respect to droplet deposition and virus transport within the lungs as well as viral infection characteristics. Droplet deposition computed using the present model is compared with the experimental data of Heyder et al. (1986) for whole lung deposition as well as alveolar deposition (see Supplementary Figure S2).

**FIGURE 1:**
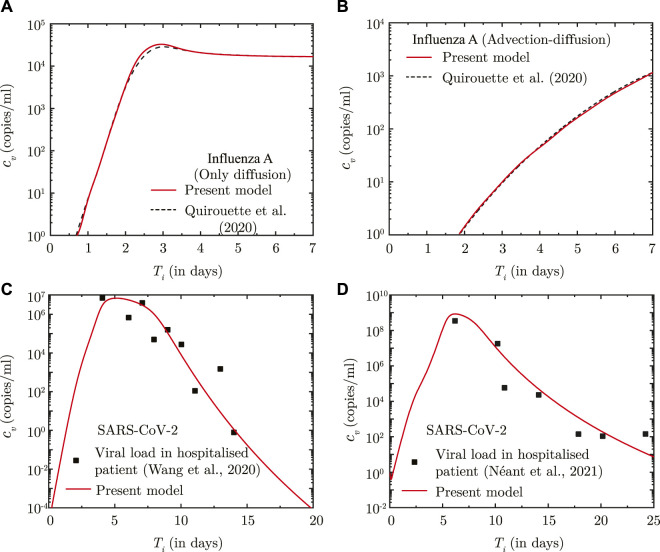
**(A, B)** Comparison between the results obtained using the present model and Quirouette et al. (2020) for Influenza A infection with respect to temporal change in spatially-averaged viral load in presence of **(A)** virus diffusion only and **(B)** considering combined virus diffusion and mucociliary advection. **(C, D)** Comparison of temporal change in the dimensional viral load (*c*_*v*_) predicted using the present infection kinetics-immune response model for patients hospitalised with SARS-CoV-2 infection with clinically determined viral load from **(A)** lower respiratory tract (Wang et al., 2020) and **(B)** nasopharyngeal region (Néant et al., 2021). *T*_*i*_ denotes time (in days) post onset of infection.

The predictions of the coupled virus transport and infection kinetics model are compared with the computational results of (Quirouette et al., 2020) for Influenza A infection (see Figures 1A, B. While only diffusive transport is considered in the comparison shown in Figure 1A, both diffusive and advective transport is taken into account for the comparison shown in Figure 1B. The immunity model is not considered in the above comparison. The corresponding model parameters are listed in Supplementary Table S3.

The infection kinetics-immunity model is additionally fitted against viral load data for SARS-CoV-2 infections. The best fits of the model are shown in Figures 1C, D for two different sets of clinically-obtained data from hospitalised patients (Wang et al., 2020; Néant et al., 2021). The best-fit parameters are listed in Supplementary Table S3. These parameters are used in this study for predicting SARS-CoV-2 viral progression. A comparison, similar to that carried out for Influenza-A, could not be carried out for SARS-CoV-2 infection due to lack of sufficient data on SARS-CoV-2 virus transport within the lungs.

It can, thus, be observed from these comparisons that the present model can appreciably determine droplet transport and deposition within the lungs, and simultaneously predict virus transport within the lungs considering the effects of infection kinetics.

## 3 Results

The only source of virus within the LRT are the droplets inhaled into the trachea from the pharynx. These may be the droplets inhaled from the environment (which have not deposited in the URT) or those formed due to ANM. In either case, these droplets are transported with airflow deeper into the LRT during which they may also get deposited in the respiratory mucosa. The viruses, thus deposited, diffuse in the mucosa and are also transported upstream by advective mucociliary clearance. At the same time, the deposited viruses undergo replication forming virus colonies and are also simultaneously removed by various mechanisms (Wiersinga et al., 2020).

In order to identify the dynamics of virus transport and infection progression in the LRT, simulations were carried out using the validated mathematical model assuming that virus-laden droplets are inhaled into the trachea for five breaths (*τ*_exp_ = 5). Extrapolation to longer exposure times and its impact on infection will be discussed separately. It is observed that the virus concentration in the mucus (*ϕ*_*v*_), at the end of the inhalation, qualitatively follows droplet deposition characteristics (see Supplementary Figure S2) due to the significantly longer time-scale of virus infection and washout, as compared to droplet deposition.

**FIGURE 2:**
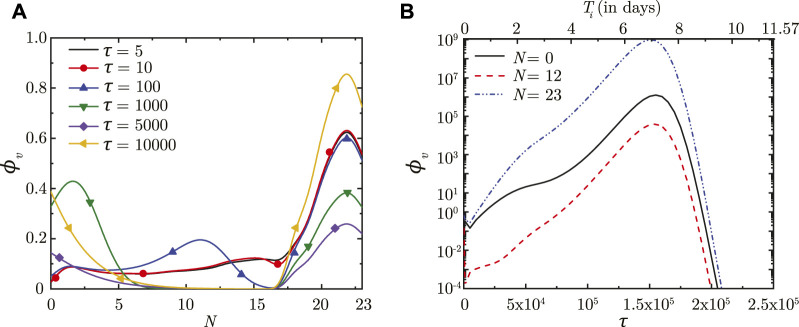
**(A)** Dimensionless virus concentration in mucus (*ϕ*_*v*_) within the LRT at different dimensionless time instances (*τ*) post onset of infection and **(B)** Temporal change in *ϕ*_*v*_ at different spatial locations within the LRT (*N* = 0, 12, 23; *N* represents the lung generation number). The results in **(B)** are shown with respect to a dimensionless time (*τ*) as well as a dimensional time (*T*_*i*_) post infection onset. The breathing time period (*T*_*b*_ = 4s) is considered for obtaining the dimensional time (see Eq. 7). All results are shown considering the baseline parameter values (see Table 1).

Once droplet inhalation ceases, viruses deposited in the upper airways of the LRT (*N* < 18) are transported upstream towards the trachea (*N* = 0) by mucociliary advection and are eventually washed out. This is evident from the spatial change in *ϕ*_*v*_ with time (see Figure 2A). The larger *ϕ*_*v*_ in the upper airways (lower *N*) is due to smaller mucus volume in those regions. The viruses continue to be washed out of the lungs as long as washout dominates over virus replication. After a certain time period (*τ* ∼ 5,000), virus replication starts to dominate over washout causing *ϕ*_*v*_ to again increase with time, as can be observed in Figure 2A. This is also corroborated by the temporal change in *ϕ*_*v*_ (see Figure 2B).

In contrast, viruses deposited in the deep lung (*N* ≥ 18) are transported only through diffusion due to absence of mucociliary advection. This leads to longer persistence of viruses deposited in the deep lung. The dynamics of *ϕ*_*v*_ in the deep lung is, thus, determined by the weak diffusive transport and virus kinetics only. It can be observed that *ϕ*_*v*_ reduces considerably in the initial period post-deposition due to larger virus clearance as compared to virus replication. Once virus replication starts to dominate, *ϕ*_*v*_ starts to increase as time progresses (see Figure 2A). The increase of *ϕ*_*v*_ at various lung generations continues as long as the impact of virus replication remains stronger than virus clearance (see Figure 2B). However, it is observed that beyond a certain *ϕ*_*v*_, virus clearance becomes stronger than replication leading to a reduction in *ϕ*_*v*_ with time. The critical virus concentration (*ϕ*_*v*, max_), thus obtained, corresponds to the peak infectious state. The corresponding time is denoted as *τ*_inf,*p*_.

Longer residence time of viruses in the deep lung allows greater replication leading to substantially higher peak *ϕ*_*v*_ (see Figure 2B). This increases the probability of severe infection including pneumonia and acute respiratory distress syndrome (ARDS). Additionally, the thin surfactant layer in the deep lungs increases the possibility of the deposited viruses entering the blood stream in the alveolated bronchioles causing viremia. Thus, it is important to understand the various conditions that cause deposition of viruses in the deep lung as well as to study the impact of these conditions on infection progression in the deep lung. This is discussed in the following sections. Physiologically relevant ranges are chosen for all parameters in this study (see Supplementary Table S3 for more details). The corresponding dimensionless parameters are summarized in Table 1 with the baseline magnitudes and the range over which they are studied.

**TABLE 1:** Baseline values of various dimensionless parameters and their ranges.

Parameter	Baseline value	Range
*Pe*_*d*_	1.32 × 10^10^	1.32 × 10^10^–1.54 × 10^12^
*Pe*_*v*_	2.28 × 10^8^	2.28 × 10^7^–2.28 × 10^8^
*St*_*a*_	.0095	.005–0.1
*St*_*m*_	359.7122	100–1,500
*τ*_exp_	5	5–10,000
*p*_0_	5.62 × 10^17^	3.8 × 10^16^–5.62 × 10^17^
*c*_*l*_	3.79 × 10^7^	0–3.79 × 10^8^
*τ*_*E*_	3 × 10^3^	Invariant
*τ*_*I*_	7.2 × 10^4^	Invariant
*f*	0.5	.2–1
*τ*_*p*,*i*_	6.48 × 10^4^	2.16 × 10^4^–19.44 × 10^4^
*λ*_*g*,*i*_	9.26 × 10^–5^	Invariant
*λ*_*d*,*i*_	4.63 × 10^–5^	Invariant
$k_{A}^{\prime }$	9.1 × 10^7^	0–9.1 × 10^8^
*Ab*_0_	.002	.001–.005
*λ*_*g*,*a*_	3.472 × 10^–5^	Invariant
$k_{C}^{\prime }$	.56 × 10^–3^	0–10^−3^
*τ*_*p*,*t*_	17.28 × 10^4^	8.64 × 10^4^–34.56 × 10^4^
*λ*_*g*,*t*_	9.26 × 10^–5^	Invariant
*λ*_*d*,*t*_	4.63 × 10^–6^	Invariant

The dimensionless magnitudes are obtained based on the corresponding dimensional parameters obtained from various sources (see Tables 2, 3 as well as Supplementary Table S1) (Weibel, 1963; Hofmann, 2011; Karamaoun et al., 2018; Mittal et al., 2020; Wiersinga et al., 2020).

### 3.1 Effect of inhaled droplet size, airflow rate and exposure duration

**FIGURE 3:**
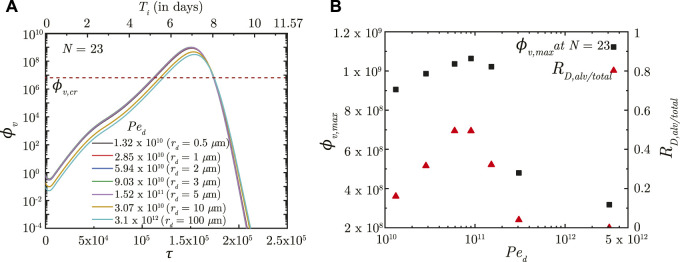
**(A)** Temporal change in dimensionless virus concentration (*ϕ*_*v*_) at *N* = 23 (deep lung) for different *Pe*_*d*_ with respect to dimensionless time (*τ*) as well as a dimensional time (*T*_*i*_) post infection onset. The breathing time period (*T*_*b*_ = 4s) is considered for obtaining the dimensional time (see Eq. 7). Dimensional droplet radii (*r*_*d*_) is additionally mentioned for each *Pe*_*d*_ considering a tidal volume of 1,000 mL. **(B)** Change in peak dimensionless virus concentration (*ϕ*_*v*, max_) in the deep lung with varying *Pe*_*d*_ (10^10^ − 5 × 10^12^; *r*_*d*_ = .4 − 160 *μ*m). Results indicate that *ϕ*_*v*, max_ in the deep lung follows a similar trend as the ratio of deep lung (alveolar) deposition to total droplet deposition in the lungs (*R*_*D*,*alv*/*total*_). The dotted line indicates the critical viral load (*ϕ*_*v*,*cr*_) in the deep lung required for pneumonia onset (Fajnzylber et al., 2020; Wölfel et al., 2020).

The effects of inhaled droplet size and airflow rate are studied by varying the droplet Peclet number (*Pe*_*d*_). *Pe*_*d*_ is defined as the ratio of advective airflow in the lung to diffusive transport of the droplets in air (see Eq. 2). As such, a stronger advective airflow or a larger inhaled droplet size results in a larger *Pe*_*d*_ and *vice versa*. Although it is expected that a larger *Pe*_*d*_ would make the inhaled droplets reach deeper regions of the lung, analyses have established that droplet deposition in the deep lung is non-monotonic (Hofmann, 2011; Chakravarty et al., 2022). The initial *ϕ*_*v*_ (and subsequently, *ϕ*_*v*, max_) in the deep lung follows the droplet deposition characteristics (see Figures 3A, B). However, the infection progression remains qualitatively similar for all *Pe*_*d*_ with no significant difference in *τ*_inf,*p*_. The time taken for infection resolution also remains unaffected since *Pe*_*d*_ does not influence virus clearance from the lung. This suggests the inevitability of an infection (which often becomes severe) even if a small fraction of the inhaled droplets reach the deep lung. Transport of viruses to the deep lung, thus, needs to be minimised.

**FIGURE 4:**
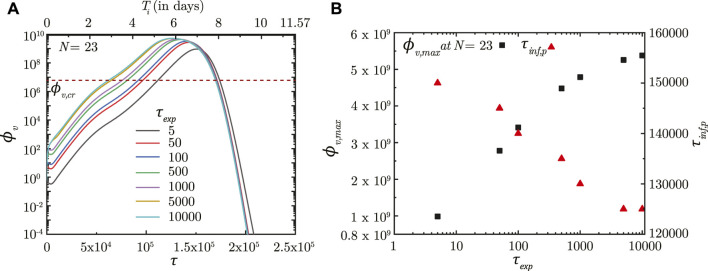
**(A)** Temporal change in dimensionless virus concentration (*ϕ*_*v*_) at *N* = 23 (deep lung) for different exposure durations (*τ*_exp_) with respect to dimensionless time (*τ*) as well as a dimensional time (*T*_*i*_) post infection onset. The breathing time period (*T*_*b*_ = 4s) is considered for obtaining the dimensional time (see Eq. 7). **(B)** Change in peak dimensionless virus concentration (*ϕ*_*v*,max_) and time required for reaching peak infection (*τ*_inf,*p*_) in the deep lung with varying *τ*_exp_. The dotted line indicates the critical viral load (*ϕ*_*v*,*cr*_) in the deep lung required for pneumonia onset (Fajnzylber et al., 2020; Wölfel et al., 2020).

In contrast, *τ*_exp_ is observed to significantly influence infection progression in the lungs although it does not influence the deposition location. A longer *τ*_exp_ results in the droplets being inhaled into the LRT for a longer duration resulting in larger droplet deposition in the mucus (Chakravarty et al., 2022) and in a higher initial *ϕ*_*v*_. This leads to faster infection progression (shorter *τ*_inf,*p*_) and a substantially larger *ϕ*_*v*, max_ (see Figure 4). Thus, minimizing the inhalation duration can help control the severity of the infection.

### 3.2 Effect of virus size, mucus advection and viscosity

The impact of virus size, mucociliary advection and mucus viscosity is studied by varying the virus Peclet number (*Pe*_*v*_). *Pe*_*v*_ is defined as the ratio of advective mucociliary transport and diffusive transport of the deposited viruses in the mucus layer (see Eq. 7). A larger *Pe*_*v*_, as such, indicates stronger mucociliary advection (or weaker diffusive transport) and *vice versa*. Weaker diffusive transport can be the result of a larger virus or more viscous mucus.

**FIGURE 5:**
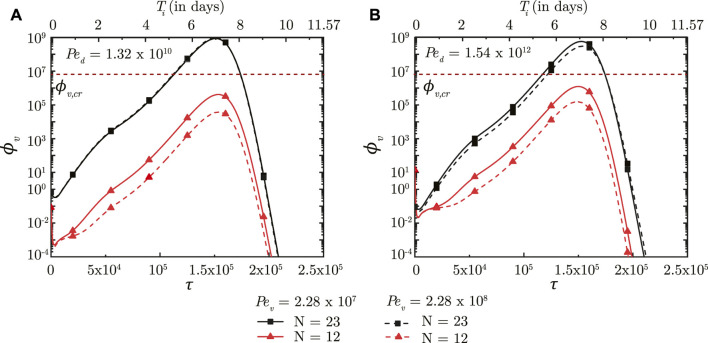
Temporal change in dimensionless virus concentration (*ϕ*_*v*_) in the upper airways (*N* = 12) and the deep lung (*N* = 23) for different *Pe*_*v*_ with respect to dimensionless time (*τ*) as well as a dimensional time (*T*_*i*_) post infection onset. The breathing time period (*T*_*b*_ = 4s) is considered for obtaining the dimensional time (see Eq. 7). The results are shown for two different *Pe*_*d*_ in **(A,B)**. The dotted line indicates the critical viral load (*ϕ*_*v*,*cr*_) in the deep lung required for pneumonia onset (Fajnzylber et al., 2020; Wölfel et al., 2020).

A larger *Pe*_*v*_ (stronger mucociliary advection) leads to relatively faster washout of viruses from the upper airways of the lung where mucociliary clearance is substantial (see Figure 5, *N* = 12). Faster washout reduces the residence time of the viruses in the upper airways. This inhibits virus replication and reduces *ϕ*_*v*, max_ in the upper airways. Similar effects are observed in the upper airways for the entire range of *Pe*_*d*_ considered in this analysis. In contrast, only a minor impact is observed in the deep lung (*N* = 23) for the range of *Pe*_*v*_ considered. This can be primarily attributed to the negligible mucociliary clearance from the deep lung. A change in *Pe*_*v*_ in the deep lung, thus, implies modification of virus diffusivity. However, the time-scale of virus diffusion is too long for it to have any substantial impact on the infection time-course in the deep lung.

While the size of inhaled viruses cannot be controlled, the rate of mucociliary clearance and mucus viscosity can be therapeutically modified. This provides a viable approach for controlling infection progression in the upper airways of the lung. A similar viable approach for deep lung infection is, however, not feasible.

### 3.3 Effect of breathing rate

The impact of breathing time period is studied by varying two parameters—the airway Strouhal number (*St*_*a*_) and the mucus Strouhal number (*St*_*m*_). *St*_*a*_ is defined as the ratio of time scale of advective airflow in the LRT to the breathing time period (see Eq. 2). *St*_*m*_ is defined as the ratio between time scale of mucociliary advection and the breathing time period (see Eq. 7). While *St*_*a*_ affects the initial deposition of the inhaled virus-laden droplets, *St*_*m*_ influences the washout of the deposited viruses and hence, the infection time-course.

**FIGURE 6:**
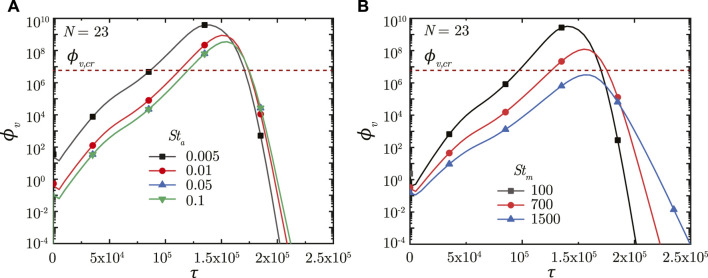
Temporal change in dimensionless virus concentration (*ϕ*_*v*_) at *N* = 23 (deep lung) for various **(A)** *St*_*a*_ and **(B)** *St*_*m*_. The dotted line indicates the critical viral load (*ϕ*_*v*,*cr*_) in the deep lung required for pneumonia onset (Fajnzylber et al., 2020; Wölfel et al., 2020).

Figure 6A shows the impact of *St*_*a*_ on *ϕ*_*v*_ in the deep lung. A longer breathing time period (smaller *St*_*a*_) leads to a larger volume of droplets inhaled into the lung, keeping all other parameters constant. This allows a greater portion of the inhaled droplets to reach the deep lung (Chakravarty et al., 2022) which, in turn, results in increased deposition of viruses. A larger *ϕ*_*v*_ at the end of *τ*_*inh*_ causes faster infection progression in the lung (shorter *τ*_inf,*p*_) and a corresponding higher *ϕ*_*v*, max_ (see Figure 6A). However, the time required for infection resolution remains similar. It is to be noted that there is no substantial difference between the results obtained in the deep lung for *St*_*a*_ ≥ .05 since droplet deposition in the deep lung remains almost invariant beyond this limit (Chakravarty et al., 2022).

A longer breathing period also reduces *St*_*m*_ suggesting greater mucociliary clearance from the upper airways in a breathing cycle, keeping all other parameters constant. In other words, less number of breathing cycles are required to achieve equivalent mucus clearance (and hence, virus washout) from the upper airways of the lung at lower *St*_*m*_. This can be observed from Figure 6B. The actual time taken for virus washout is, however, much longer since *St*_*m*_ is inversely dependent on *T*_*b*_. Thus, faster virus washout takes place from the upper airways at larger *St*_*m*_ and *vice versa*. This leads to a steeper concentration gradient between the deep lung and the upper airways resulting in faster diffusive transport in the deep lung. Thus, infections get resolved relatively faster in the deep lung at larger *St*_*m*_. In addition, a larger *T*_*b*_ (for lower *St*_*m*_) also allows the deposited viruses to replicate more leading to relatively higher *ϕ*_*v*, max_ at lower *St*_*m*_ (see Figure 6B).

In summary, longer breaths result in a larger *ϕ*_*v*, max_, and slower infection resolution, both of which are bad from a clinical perspective. *ϕ*_*v*, max_ beyond a certain limit in the deep lung (see Figure 6B) can cause pneumonia, which may become life threatening. Thus, shorter breaths, which lowers the viral load in the lung and relatively shortens the infection time-course, are more beneficial.

### 3.4 Effect of virus growth and clearance rates

**FIGURE 7:**
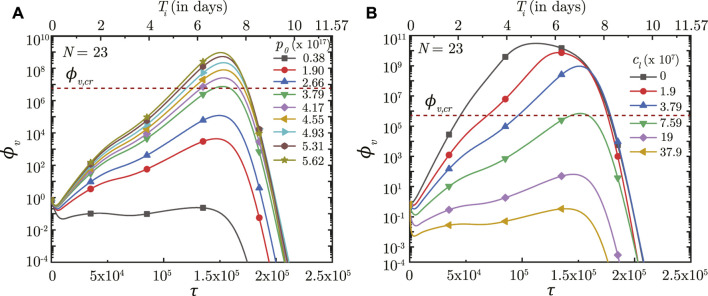
Temporal change in dimensionless virus concentration (*ϕ*_*v*_) at *N* = 23 (deep lung) for different **(A)** virus replication rates (*p*_0_) and **(B)** virus clearance rates (*c*_*L*_). The results are shown with respect to dimensionless time (*τ*) as well as a dimensional time (*T*_*i*_) post infection onset with the breathing time period (*T*_*b*_ = 4s) considered for obtaining the dimensional time (see Eq. 7). The dotted line indicates the critical viral load (*ϕ*_*v*,*cr*_) in the deep lung required for pneumonia onset (Fajnzylber et al., 2020).

The impact of virus growth and clearance is studied by varying *p*_0_ and *c*_*l*_, respectively, other parameters remaining fixed. It is observed that a higher *p*_0_ (higher virus replication) leads to larger *ϕ*_*v*_ in the deep lung throughout the time-course of infection (see Figure 7A). *ϕ*_*v*, max_ in the deep lung, as such, increases as *p*_0_ becomes larger and *vice versa*. However, no significant change is observed in *τ*_inf,*p*_.

In contrast, the virus clearance rate (*c*_*L*_) is observed to affect *ϕ*_*v*_ as well as *τ*_inf,*p*_ (see Figure 7B). Greater *c*_*l*_ results in larger virus clearance from the lung which reduces *ϕ*_*v*_ (and hence, *ϕ*_*v*, max_) and also delays attainment of the peak infectious state. The reverse happens when *c*_*l*_ is reduced.

### 3.5 Effect of immunity

The simplified immune response considered in the present study is a combined model of humoral response (antibodies), innate response (interferons) and cellular response (cytotoxic T-lymphocytes). As such, it becomes necessary to distinguish between these responses in order to identify the key effects of the individual responses on virus concentration in the lung and infection time-course. This is achieved by varying the parameters of a specific response model, while keeping the other response models constant.

#### 3.5.1 Antibody (humoral) response

**FIGURE 8:**
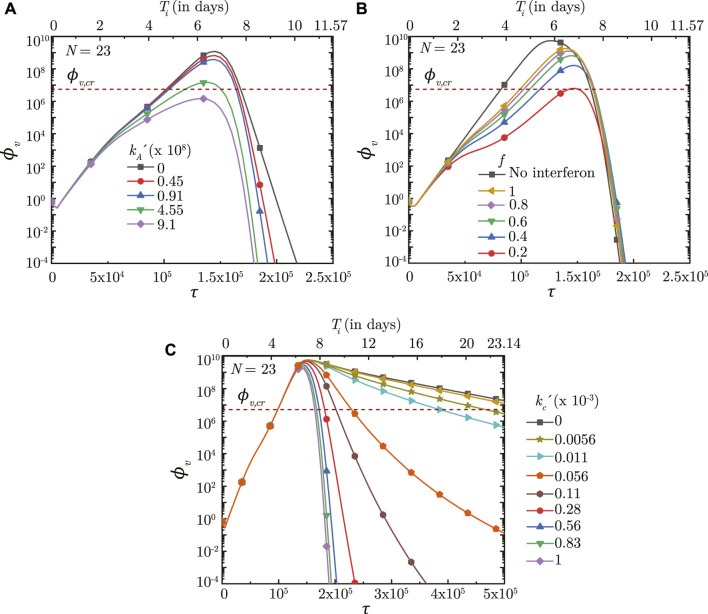
Temporal change in dimensionless virus concentration (*ϕ*_*v*_) at *N* = 23 (deep lung) with change in **(A)** binding affinity of antibodies $\left(k_{A}^{\prime }\right)$ , **(B)** interferon requirement for halving virus production (*f*), and **(C)** rate at which cytotoxic T-lymphocytes eliminate the infected cells $\left(k_{c}^{\prime }\right)$ . The results are shown with respect to dimensionless time (*τ*) as well as a dimensional time (*T*_*i*_) post infection onset with the breathing time period (*T*_*b*_ = 4s) considered for obtaining the dimensional time (see Eq. 7). The dotted line indicates the critical viral load (*ϕ*_*v*,*cr*_) in the deep lung required for pneumonia onset (Fajnzylber et al., 2020).

**FIGURE 9:**
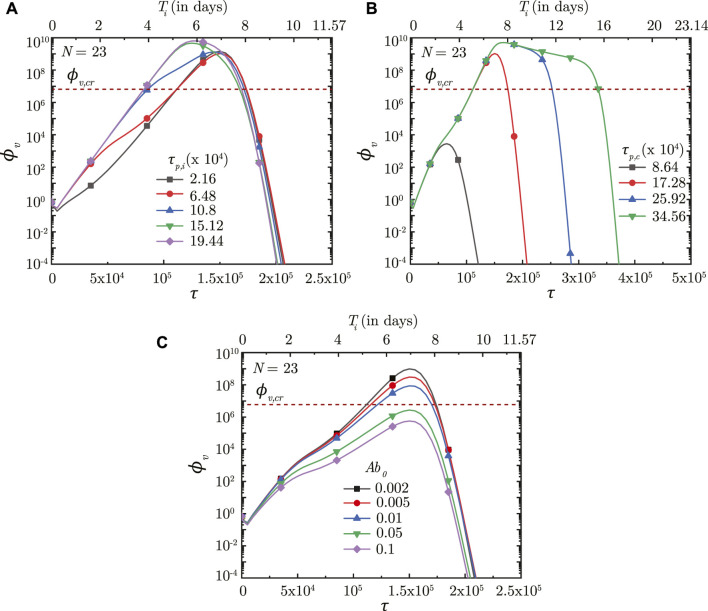
Temporal change in dimensionless virus concentration (*ϕ*_*v*_) at *N* = 23 (deep lung) with change in the time required for **(A)** interferon (*τ*_*p*,*i*_) and **(B)** cytotoxic T-lymphocytes (*τ*_*p*,*c*_) to reach their peak levels, and **(C)** with change in the initial antibody level (*Ab*_0_) in the infected individual. The results are shown with respect to dimensionless time (*τ*) as well as a dimensional time (*T*_*i*_) post infection onset with the breathing time period (*T*_*b*_ = 4s) considered for obtaining the dimensional time (see Eq. 7). The dotted line indicates the critical viral load (*ϕ*_*v*,*cr*_) in the deep lung required for pneumonia onset (Fajnzylber et al., 2020).

The humoral immune response mechanism acts by producing antibodies in response to an infection (or vaccination). These antibodies bind with the pathogens (viruses) and neutralize them, thereby enhancing the virus clearance rate. The efficacy of this response is, thus, dependent on the rate at which the antibodies neutralize the pathogens $\left(k_{A}^{\prime }\right)$ and the amount of antibodies present in the body (which is a function of the initial antibody concentration *Ab*_0_ and the antibody growth rate *λ*_*g*,*α*_). Results (see Figures 8A, 9C) indicate that the initial infection time-course remains similar irrespective of $k_{A}^{\prime }$ and *Ab*_0_. The antibody concentration builds up over time and it is only after the antibody concentration becomes large enough that the impact on infection becomes apparent. A larger $k_{A}^{\prime }$ (greater virus neutralization) is observed to cause faster clearance of viruses from the lung (see Figure 8A). Similarly, a larger *Ab*_0_ (more antibody availability) allows initial targeting of greater number of viruses which, in turn, reduces the virus replication. This results in a lower *ϕ*_*v*, max_ and relatively shorter *τ*_inf,*p*_ (see Figure 9C). The infection also gets resolved within relatively short periods.

#### 3.5.2 Interferon (innate) response

The innate immune response mechanism works by production of interferons which inhibits virus replication through various secondary mechanisms. The effects of such secondary mechanisms are not considered in detail in the present model and the sole effect of interferons is modeled to be reduction in virus growth rate (*p*_0_). Figure 8B shows the impact of interferons (through the parameter *f*) on the infection time-course in the deep lung. *f* is defined such that, for *f* = .5, the virus growth rate is halved when interferon concentration is at 50% of its peak concentration. A larger *f*, therefore, has a smaller impact on the virus concentration in the lung and *vice versa*. *ϕ*_*v*, max_ is also observed to reduce significantly and *τ*_inf,*p*_ becomes relatively longer with decreasing *f*.

The time taken for interferon concentration to reach its peak (*τ*_*p*,*i*_) is also observed to influence the infection time-course (see Figure 9A). Early peaking of interferons (lower *τ*_*p*,*i*_) reduces *ϕ*_*v*_ during the initial stages of infection, but does not have any significant influence during the later stages. As *τ*_*p*,*i*_ becomes longer, the impact on virus production during the initial stages of infection becomes delayed resulting in relatively larger *ϕ*_*v*_. Beyond a certain *τ*_*p*,*i*_, however, no substantial change is observed on the virus concentration.

#### 3.5.3 *T*-lymphocyte (cellular) response

The cellular response mechanism is dependent on the action of cytotoxic *T* − lymphocytes on the infected cells. The *T* − lymphocytes act by recognising and neutralising the infected cells. The impact of the cellular response mechanism is, thus, dependent on the rate at which the *T* − lymphocytes neutralise the infected cells $\left(k_{C}^{\prime }\right)$ and also on the *T* − lymphocytes concentration (*T*_*L*_) in the body.

Results indicate that the impact of *T* − lymphocytes on *ϕ*_*v*_ remains negligible during the initial period of infection mainly due to low *T*_*l*_ in the body (see Figures 8C, 9B). *T*_*l*_ builds up as time progresses causing larger neutralisation of the infected cells (see Eq. 15). This, in turn, reduces *ϕ*_*v*_ especially during the later stages of infection. The rate at which *ϕ*_*v*_ reduces during this period is observed to vary substantially with $k_{C}^{\prime }$ . A larger $k_{C}^{\prime }$ results in a faster decrease in *ϕ*_*v*_ leading to early infection resolution and *vice versa* (see Figure 8C). It is interesting to note that the infections become prolonged when the neutralization rates become abnormally low $\left(k_{C}^{\prime }<0.05\right)$ highlighting the importance of *T* − lymphocytes in fighting viral infections.

Figure 9B shows the effect of *T*_*l*_ in the body (in terms of *τ*_*p*,*t*_) on *ϕ*_*v*_ in the deep lung. *τ*_*p*,*t*_ is defined as the time taken by *T*_*l*_ to reach its peak. A shorter *τ*_*p*,*t*_ results in faster buildup of *T*_*l*_ and *vice versa* (see Eq. 15). Faster buildup of *T*_*l*_ allows the cellular response mechanism to start acting early which, in turn, restricts *ϕ*_*v*, max_ to small magnitudes and also causes early infection resolution. The reverse happens when *τ*_*p*,*t*_ becomes longer thereby highlighting the need of having *T* − lymphocytes present in the body prior to infection.

## 4 Discussion

### 4.1 Variant to variant difference

One of the key aspects of the SARS-CoV-2 pandemic has been the consistent mutation of the virus leading to emergence of newer variants as the pandemic progressed. Some of these variants (for e.g., Omicron) caused faster infection spread with milder symptoms in the infected individuals. Other variants (for e.g., Delta) often caused more severe health effects (Puhach et al., 2022; Silva et al., 2022). The time-course of infection also varied from one variant of the virus to the other. Symptoms lasted typically for ∼7 days in case of the Omicron variant as compared to ∼9 days for the Delta variant (Menni et al., 2022).

Our results indicate that the reason for such difference in symptom severity and infection time-course can be correlated to the relative replication and neutralization of the virus in the host cells (see Figure 7). For a given rate of virus neutralization, the viral load reduced and the infection time-course shortened as the replication rate attenuated. Greater virus neutralization rate, for a given virus replication rate, has a similar impact. A lower viral load suggests occurrence of milder symptoms in the infected individual. Thus, it can be stated that the virus replication rate in the lung becomes attenuated (or virus neutralization is enhanced) in case of the variants causing milder symptoms and shorter infection time-courses (for e.g., Omicron) as compared to the variants causing severe symptoms and longer infection time-courses (for e.g., Delta). A recent study by Silva et al. (2022) corroborates this inference.

### 4.2 Vaccination and acquired immunity

Vaccination against SARS-CoV-2 has played a major role in suppressing the severity of the pandemic. Vaccines work by inducing the production of *B* − lymphocytes and *T* − lymphocytes (or memory cells) within the body (Scourfield et al., 2021; Zollner et al., 2021). The *B* − lymphocytes are responsible for the production of antibodies which fight the viruses. *T* − lymphocytes are cytotoxic in nature and attack the cells infected by the virus. The end outcome is that the body is left with heightened defenses against the virus. Similar effects are true for prior infections as well, although magnitudes of such effects may vary.

Our results confirm that prior presence of *T* − lymphocytes within the body readily suppresses an infection (see Figure 9B). Prior presence of *T* − lymphocytes (which reduces the time required for *T* − lymphocyte concentration to peak) suppress virus replication from the onset of infection itself leading to lower *ϕ*_*v*, max_ and hence, reduces the probability of disease severity. Similarly, a larger initial antibody presence in the body allows targeting (and subsequent neutralisation) of a larger number of viruses. This slows down virus replication and enables other immune effects to act effectively, thereby decreasing disease severity (see Figure 9C).

Effectiveness of the antibodies and *T* − lymphocytes also play an important role in infection progression. An effective antibody against SARS-CoV-2 neutralizes the viruses at a much faster rate, thereby, restricting its replication and reducing probability of the infection progressing to a severe disease (see Figure 8A). Effectiveness of the *T* − lymphocytes, however, does not influence infection progression until their concentration becomes large enough, after which an effective *T* − lymphocyte readily suppress the infection (see Figure 8C).

### 4.3 Deep lung infection in SARS-CoV-2

Results indicate that a certain portion of virus-laden droplets present at the entrance to the trachea inevitably reach the deep lung where they deposit releasing the viruses causing infection in the deep lung. These droplets are a combination of the inhaled droplets (which have not deposited in the nasopharynx) and those formed due to aerosolisation of the mucus layer during respiratory motions (coughs etc.). More the availability of droplets, longer is the duration for which these may be inhaled into the LRT and greater is the probability of these droplets reaching the deep lung causing infection (see Figure 4). Clinical conditions such as bronchitis, asthma, etc. accentuate the probability of formation of aerosolised droplets due to mucus hypersecretion or airway constriction (Fahy and Dickey, 2010). Thus, the probability of contracting deep lung infection remains higher when clinically associated with other upper airway diseases (Lee et al., 2020) which promote aerosolization of nasopharyngeal mucus.

The severity of a deep lung infection is determined by the binding affinity of the virus with cells in the deep lung as well as the replication/neutralization characteristics of the virus. Binding affinity is lower and replication becomes attenuated for the Omicron variant of SARS-CoV-2 in the deep lung cells (Silva et al., 2022) resulting in much lower probability of a severe deep lung infection. This is in contrast to the Delta variant for which the probability of a severe deep lung infection is much more due to its higher binding affinity and larger replication rate in the cells of deep lung (Silva et al., 2022). Greater the severity of deep lung infection, larger is the probability of development of pneumonia and associated respiratory complications.

### 4.4 Time-scale of pneumonia onset in a SARS-CoV-2 infection

Onset of pneumonia is a common yardstick used to gauge the severity of any infection since it can progress rapidly to respiratory failure. Clinical studies have found that the time taken for the development of respiratory failure from pneumonia in a human body from a SARS-CoV-2 infection is typically 6–14 days after the initial onset of symptoms (Feng et al., 2020; Grant et al., 2021), primarily after oropharyngeal infection (Wiersinga et al., 2020). There is autopsy-based evidence of the migration of deposited viruses from the pharyngeal region to the lower respiratory tract and the distal alveolar region (Hou et al., 2020; Grant et al., 2021), although the mechanism still remains unclear. Aspiration is a possible mechanism Basu et al. (2022). Another plausible mechanism is through inhalation of infected droplets as considered in the present study. Residual inhaled droplets or those formed by aerosolisation of the infected mucosa (due to interaction of the infected mucosa with airflow) are subsequently transported from the nasopharynx deeper into the lung where they can release the viruses causing further infection (Darquenne et al., 2022).

The results presented above are used to estimate the time taken for the SARS-CoV-2 concentration in the deep lung to reach the critical magnitude (*c*_*v*,*cr*_) required for onset of pneumonia. Virus concentration in the pharyngeal region (*N* = 0) is assumed to be the clinically observed viral load in mild infections in pharyngeal samples, where SARS-CoV-2 is initially registered (*c*_*v*,0_ = 10^2^ copies/mL) (Fajnzylber et al., 2020). The critical concentration in the deep lungs (*c*_*v*,*cr*_) is assumed to be the clinically observed median viral load in sputum samples (7 × 10^6^ copies/mL) of symptomatic individuals exhibiting pneumonia (Fajnzylber et al., 2020; Wölfel et al., 2020) (see Supplementary Tables S3, S4 for time-estimates with other *c*_*v*,*cr*_). It is further assumed that the pharyngeal viral load remains constant throughout the inhalation duration. It is calculated that the time required for onset of pneumonia in a SARS-CoV-2 infection can vary from ∼2.5−7 days, depending on *c*_*v*,*cr*_ and associated parameters. Table 2 summarises the change in pneumonia onset time with variation in different fluid dynamics and physiological parameters. Table 3 lists the change in pneumonia onset time with variation in different infection parameters.

**TABLE 2:** Variation in estimated time required for onset of SARS-CoV-2 pneumonia with change in various dimensional fluid dynamic and physiological parameters.

Parameter	Magnitude	Estimated pneumonia
		onset time (days)
*d*_*d*_ (*μ*m)	100	5.65
	10	5.56
	5	5.23
	3	5.12
	2	5.14
	**1**	5.19
	.5	5.23
*d*_*v*_ (nm)	10	5.25
	**100**	5.19
*T*_*b*_ (s)	1	5.67
	2	5.78
	**4**	5.19
	8	3.52
*Q*_0_ (m^3^/s)	7.088 × 10^–4^	5.37
	**7.875 × 10** ^**–4**^	5.19
	8.66 × 10^–4^	5.09
*L*_0_ (m)	.108	5.09
	**.12**	5.19
	.132	5.32
*T*_exp_ (s)	**20**	5.19
	200	4.54
	400	4.35
	1,000	3.84
	4,000	3.56
	20,000	3.05
	40,000	2.96

The results are shown considering *c*_*v*,*cr*_ = 7 × 10^6^ copies/mL in the deep lung (Fajnzylber et al., 2020; Wölfel et al., 2020). The following baseline parameters are used (highlighted in boldface): *d*_*d*_ = 1 *μ*m, *d*_*v*_ = 100 nm, *T*_*b*_ = 4s, *Q*_0_ = 7.875 × 10^–4^ m^3^/s, *L*_0_ = .12 m, *T*_*inh*_ = 20s (Weibel, 1963; Hofmann, 2011; Mittal et al., 2020; Wiersinga et al., 2020).

**TABLE 3:** Variation in estimated time required for onset of SARS-CoV-2 pneumonia with change in various dimensional infection parameters.

Parameter	Physiological effect	Contributing factors	Magnitude	Estimated pneumonia
				onset time (days)
*p*	A larger *p* enhances virus replication in the infected cells	Virus-cell interaction	7 × 10^10^	No pneumonia onset
			1.0 × 10^11^	6.95
			1.1 × 10^11^	6.25
			1.2 × 10^11^	5.88
			1.3 × 10^11^	5.61
			1.4 × 10^11^	5.42
			**1.48 × 10** ^**11**^	5.19
*c*	A larger *c* enhances virus clearance from the body	Virus-cell interaction	0	2.55
			5	3.98
			**10**	5.19
			20	No pneumonia onset
*f*	A smaller *f* suppresses virus replication	Effectiveness of the interferons	No Interferon	3.89
			1	4.63
			0.8	4.72
			0.6	4.95
			**0.5**	5.19
			.4	5.42
			.2	No pneumonia onset
*t*_*p*,*i*_	A shorter *t*_*p*,*i*_ indicates faster interferon buildup in the body	Prior interferon shots	1	5.19
			**3**	5.19
			5	4.03
			7	3.8
			9	3.8
*A*_0_	A larger *A*_0_ indicates a greater initial amount of antibodies	Prior Infection/Vaccination	.001	5.14
			**.002**	5.19
			.005	5.46
			.01	5.64
			.05	No pneumonia onset
*k*_*A*_	A larger *k*_*A*_ enhances virus neutralization	Vaccine efficacy at producing	0	4.98
		appropriate antibodies enhances *k*_*A*_	.5	5.1
			**1**	5.19
			5	5.9
			10	No pneumonia onset
*t*_*p*,*c*_	A shorter *t*_*p*,*c*_ indicates prior presence of T-lymphocytes	Prior Infection/Vaccination	4	No pneumonia onset
			**8**	5.19
			12	5.19
			16	5.19
*k*_*C*_	A larger *k*_*C*_ enhances virus neutralization	Vaccine efficacy at producing	0	5.16
		appropriate T-lymphocytes enhances *k*_*C*_	.01	5.16
			.1	5.18
			**.5**	5.19
			1	5.19

The results are shown considering *c*_*v*,*cr*_ = 7 × 10^6^ copies/mL in the deep lung (Fajnzylber et al., 2020; Wölfel et al., 2020). The following baseline parameters are used (highlighted in boldface): *p* = 1.48 × 10^11^ virus-copies/mL/day, *c* = 10/day, *f* = .5, *t*_*p*,*i*_ = 3 days, *A*_0_ = .002 copies/mL, *k*_*A*_ = 1/h, *t*_*p*,*c*_ = 8 days, *k*_*C*_ = .5/h (Quirouette et al., 2020; Wang et al., 2020; Néant et al., 2021).

These estimates suggest no significant change (<3.5%) in pneumonia onset time with change in virus size (*d*_*v*_), volume flow rate of air (*Q*_0_) and length geometry (*L*_0_). A relatively larger change (∼8.9%) is observed with variation in droplet diameter (*d*_*d*_), while a substantial change is observed when the breathing period (∼33%) and inhalation duration (∼43%) is varied. In contrast, significant changes in the pneumonia onset time are observed with variation in most of the infection parameters. The onset time is observed to change by ∼34% and ∼51% within the studied range of virus replication rate and clearance rate, respectively. No pneumonia is observed to be established below a critical replication rate and above a critical clearance rate (see Table 3). Pneumonia onset is fastest when interferons are absent or take a long time to build up and *vice versa*, with ∼27% variation in the onset time. A large enough interferon effectiveness sufficiently suppressed virus replication to resist pneumonia onset. A larger initial antibody presence and greater effectiveness of the antibodies at neutralizing the viruses delayed pneumonia onset ( ∼10% and ∼20% variation in onset time, respectively) with no pneumonia occurrence above a critical magnitude. Interestingly, pneumonia onset time did not change significantly with change in effectiveness of the *T* − lymphocytes. However, a fast enough production of *T* − lymphocytes resisted pneumonia onset.

## 5 Summary

Respiratory viruses, such as SARS-CoV-2, are primarily transmitted through the airborne route through respiratory droplets. When a subject inhales these droplets, they are deposited in respiratory mucus of the upper respiratory tract, where the virus infects and replicates in the nasopharyngeal epithelial cells. Some patients may develop severe pneumonia and acute respiratory distress syndrome if the infection spreads to the deep lung (alveolar region). This work discusses whether a nasopharyngeal infection can spread to the deep lung through inhalation of droplets, present in the nasopharynx, into the lower respiratory tract and the degree of severity of such an infection. A coupled mathematical model of droplet and virus transport is solved computationally considering virus infection kinetics and the effect of key dimensionless parameters is investigated. Different conditions are analysed through these dimensionless parameters based on which necessary remedies and public health recommendations can be suggested.

Results indicate that fluid dynamics play an important role only in transporting the droplets from the nasopharynx to various regions of the lower respiratory tract (including the deep lung), where the droplets deposit and release the viruses causing further infection. Progress of this infection is independent of the viral load deposited. However, infection severity depends on the deposited viral load - a smaller viral load causing a milder infection and *vice versa*. Thus, adequate measures need to be adopted to prevent conditions which promote larger viral load deposition in the lower respiratory tract, particularly the deep lung. One such measure is prevention of self-aerosolization of nasopharyngeal mucus layer which provides an additional source of virus-laden droplets in the nasopharynx for further inhalation into the lower respiratory tract. Another measure is avoiding longer breaths which reduces the volume of droplets inhaled.

Once an infection initiates, growth and resolution of the infection is determined by the infection kinetics and immune responses. A larger virus replication rate (or smaller clearance rate) increases the chance of a severe infection leading to pneumonia onset, and *vice versa*. Specifically, the model predicts, in agreement with clinical observations, that a severe infection (pneumonia) can develop in the deep lung within 2.5–7 days of initial symptom onset, when nasopharyngeal droplets are inhaled into the lower respiratory tract. The immune responses, particularly antibodies and *T* − lymphocytes, are observed to be critically important for preventing infection severity and achieving quicker infection resolution. Stronger immune responses—which may be due to a prior infection or induced by vaccination—significantly lowers the chance of a severe infection. This reinforces the need of vaccination in preventing severe infections from SARS-CoV-2.

## Data Availability

The original contributions presented in the study are included in the article/Supplementary Material, further inquiries can be directed to the corresponding author.
