# Supplementary material for: Inhalation of virus-loaded droplets as a clinically plausible pathway to deep lung infection
Source: Front Physiol. 2023 Jan 19;14:1073165. doi: 10.3389/fphys.2023.1073165 (PMC9892651; doi:10.3389/fphys.2023.1073165)
Supplement: Supplementary file 1 [file DataSheet1.PDF]

Supplementary Material

1 IDEALIZATION OF THE LOWER RESPIRATORY TRACT

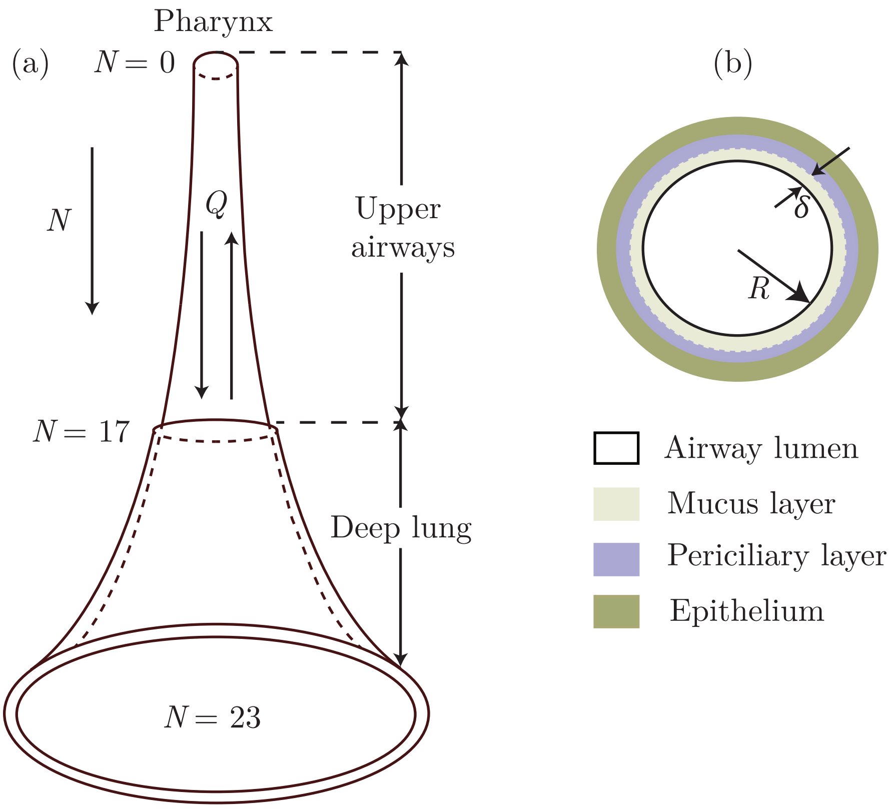

Figure S1. (a) Schematic illustration of the one-dimensional *trumpet* model that is used in the present analysis to approximate the dichotomous network structure of a human lungs (lower respiratory tract). (b) A cross-sectional view of a single airway branch is also shown to illustrate the arrangement of the airway lumen and the epithelial lining with respect to the intermediate mucus layer and the periciliary layer.

The present work utilises a one-dimensional *trumpet* model to approximate the dichotomous branching strucure of a human lung (Fig. S1). The *trumpet* model is created by subdividing the lower respiratory tract (LRT) into 24 generations ($N = 0 - 23$; N is the generation number) based on the branching structure of the lung (2^N bronchioles in each generation), where $N = 0$ and 23 corresponds to the entrance of trachea and the alveolar region, respectively. The airway is modelled as a continuous one-dimensional channel with varying dimensions at each generation. The length (L) and the total cross-sectional area (A) of the airway at each generation is calculated using a power-law function as

$$L(N) = L_0\alpha^N, A(N) = A_0(2\beta)^N, \tag{S1}$$

where L_0 and A_0 denote the length and cross-sectional area at $N = 0$, respectively (see Table S1). α and β are defined as the length-change and area-change factors, and are chosen (see Table S1) such that the length and area at each generation of the *trumpet* model closely matches Weibel's morphometric data of a human lung (Weibel et al., 1963). Alveolation of the airways is taken into account by considering an

additional area (see Table S1) in the relevant generations ($N = 17$ onwards), consistent with human lung morphology (Weibel et al., 1963). Note that although N is an integer, it is treated as a continuous variable in all transport equations for computational convenience.

Table S1. Parameters used in modelling the lung geometry

L_0	0.12 m (Weibel et al., 1963)
A_0	0.000317m^2 (Weibel et al., 1963)
R_0	$\sqrt{A_0/\pi}$
δ_0	10 μm (Karamaoun et al., 2018)
$A_{m,0}$	$2\pi R_0 \delta_0$
$V_{m,0}$	-5 mm/min (Karamaoun et al., 2018)
α	0.73
β	0.71
ζ	0.9
ε	0.87

Physiologically, the lung airways are lined with a thin layer of mucus separating it from the underlying epithelial tissue. The modeled system of airways is, thus, assumed to have a thin mucus layer lining the airway lumen and separating it from the epithelium. Mucociliary transport is also taken into consideration by assuming the mucus to be convected upwards from the deeper generations towards $N = 0$. The following power-law relations are used to estimate the thickness (δ), total cross-sectional area (A_m), and convective velocity (V_m) of the mucus layer at different lung generations -

$$\begin{aligned} \delta(N) = \delta_0 \zeta^N,\ A_m(N) = A_{m,0}(2\sqrt{\beta}\zeta)^N, \\ V_m(N) = V_{m,0}\varepsilon^N, \text{ for N} < 18, \\ = 0, \text{ for N} \geq 18, \end{aligned} \tag{S2}$$

where δ_0, $A_{m,0}$, and $V_{m,0}$ correspond to the respective magnitudes at $N = 0$ (see Table S1). ζ and ε (see Table S1) are chosen based on experimental data (Karamaoun et al., 2018). V_m is considered to be zero beyond $N = 18$ (Eq. S2) due to negligible mucus transport in the deep lungs (Mauroy et al., 2011). Note that δ and V_m are assumed to be temporally invariant in this analysis (Karamaoun et al., 2018).

Table S2 lists the assumed fraction of airway area (γ) that is alveolated at each generation in the lung model. This is required in calculating droplet deposition in the deep lung (see *S1 Text* in Chakravarty et al. (2022) for details of droplet deposition models).

Table S2. Fractions of alveolated airways in different generations (Devi, 2018)

Lung Generation (N)	Fraction of alveolated area (γ)
0-16	0
17	0.0011
18	0.0041
19	0.0135
20	0.0509
21	0.1168
22	0.2712
23	0.5424

2 DROPLET TRANSPORT MODEL

The one-dimensional transport equation for droplets in the idealised respiratory tract can be expressed as

$$\frac{\partial(Ac_d)}{\partial t} + \frac{\partial(Qc_d)}{\partial x} = \frac{\partial}{\partial x}\Big(AD_d\frac{\partial c_d}{\partial x}\Big) - L_D c_d, \tag{S3}$$

where, c_d represents the droplet concentration and D_d represents droplet diffusivity in air. Q represents the volume flow rate of air during breathing. L_D is the droplet deposition coefficient in the airway mucus. This equation is based on the *trumpet* model proposed by Taulbee & Yu (Taulbee and Yu, 1975) and has been extensively used to study different aspects of droplet deposition in the lung Devi et al. (2016); Darquenne and Paiva (1994); Mitsakou et al. (2005). It is assumed while formulating Eq. S3 that the droplets are monodispersed, do not undergo coagulation, and are decoupled from airflow in the lungs. Impact of external forces (such as electrical and magnetic forces) on droplet dynamics is assumed to be negligible. No additional source of droplets are considered to be present within the lungs and the droplets either deposit in the airway mucus or are washed out of the airways.

Equation S3 is presented in terms of airway length (x), while the respiratory tract model adopted is in terms of lung generation number (N; see Fig. S1)). As such, Eq. S3 needs to be converted to a more appropriate form in terms of N. This requires an additional mathematical relation (Eq. S4) connecting airway length x and the lung generation number (N) as follows -

$$H = \frac{\partial N}{\partial x} = -\frac{1-\alpha}{L_0\alpha\,\ln(\alpha)\alpha^N}. \tag{S4}$$

Converting Eq S3 using Eqs S4 and $A_N = A_0(2\beta)^N$, we get

$$A_0(2\beta)^N\frac{\partial c_d}{\partial t} = H\frac{\partial}{\partial N}\Bigg[\Big(A_0(2\beta)^N D_d H\frac{\partial c_d}{\partial N}\Big) - \Big(Q_{max}q(t)c_d\Big)\Bigg] - L_D c_d, \tag{S5}$$

where, $q(t)$ represents the temporal sinusoidal function accounting for airflow variation during breathing such that $Q = Q_{max}q(t)$. Eq S5 is reduced to its dimensionless form by multiplying and dividing Eq S5 with $\Big(\frac{L_0}{A_0D_d}\Big)$ and $\Big(-\frac{\alpha\,\ln(\alpha)}{1-\alpha}\Big)$, respectively, and using the following scaling parameters

$$\tau = \frac{t}{T_b}, \phi_d = \frac{c_d}{c_{d,0}}, T_a = \frac{L_0A_0}{|Q_{max}|}, St_a = \frac{T_a}{T_b}, Pe_d = \frac{|Q_{max}|L_0}{A_0D_d}, D_d = \frac{k_BTC_S}{3\pi\mu_a d_d}, \tag{S6}$$

where, Pe_d and St_a are the droplet Peclet number and airway Strouhal number, respectively. ϕ_d and τ are the dimensionless droplet concentration and dimensionless time, respectively. The quantities T_a and T_b represents the convective airflow time-scale and the breathing time-scale, respectively. The expression of D_d is based on the Stokes-Einstein relation (Chakravarty et al., 2019), where C_s represents the Cunningham slip correction, T represents the ambient temperature, μ_a denotes air viscosity, and d_d denotes the droplet diameter.

The dimensionless equation (equivalent to Eq 1 in the main manuscript), thus, obtained is used to model droplet transport in the present study and is given by

$$|Pe_d|St_a(2\alpha\beta)^N\frac{\partial(\phi_d)}{\partial\tau} = \frac{\partial F_d}{\partial N} - L'_D\phi_d, \tag{S7}$$

where, L'_D represents the dimensionless form of droplet deposition coefficient and F_d represents the total droplet flux. These are expressed as follows -

$$L'_D = L_D\frac{L_0^2}{A_0D_d}\alpha^N \tag{S8}$$

$$F_d = \left[\left(\left(\frac{2\beta}{\alpha}\right)^N\left(\frac{1-\alpha}{\alpha(\ln\alpha)}\right)^2\frac{\partial\phi_a}{\partial N}\right) + \Big(|Pe_d|q(t)\Big(\frac{1-\alpha}{\alpha\ \ln(\alpha)}\Big)\phi_d\Big)\right]. \tag{S9}$$

Different empirical models have been used to estimate droplet deposition through the major deposition mechanisms viz. sedimentation, diffusion and impaction. These can be found in detail in Chakravarty et al. (Chakravarty et al., 2022) and are not repeated here.

3 VIRUS TRANSPORT MODEL

The one-dimensional transport equation for the viruses deposited in the respiratory mucosa of the LRT can be expressed as

$$\frac{\partial(A_mc_v)}{\partial t} + \frac{\partial(Q_mc_v)}{\partial x} = \frac{\partial}{\partial x}(A_mD_v\frac{\partial c_v}{\partial x}) + \text{Source} - \text{Sink} \tag{S10}$$

where, c_v denotes the virus concentration in the mucosa, Q_m represents the volume flow rate of mucociliary clearance and D_v denotes the diffusivity of the deposited viruses in the mucosa.

The droplets deposited in the mucosa serve as the only source of viruses in the LRT. In addition, the deposited viruses replicate in the infected cells releasing new virions. The deposited and released viruses are cleared at a particular rate (c). The source term in Eq. S10 is, therefore, summation of the viruses deposited through droplets (equivalent in magnitude to the droplet deposition magnitude (L_Dc_d) times the virus load in the droplets (ϕ_l)) and new viruses released due to replication. an. Mathematically, this is expressed as -

$$\text{Source} = L_Dc_d\phi_l + pIA_m \tag{S11}$$

where, p is the virus replication rate and I is the fraction of infectious cells at a particular generation. The sink term in Eq. S10 is expressed as -

$$\text{Sink} = cc_vA_m \tag{S12}$$

Equation S10 is converted to a form in terms of N using $H = \dfrac{\partial N}{\partial x} = \dfrac{1-\alpha}{L_0\alpha\ln(\alpha)\alpha^N}$ and $A_m = A_{m,0}(2\sqrt{\beta}\zeta)^N$ as follows -

$$A_{m,0}(2\zeta\sqrt{\beta})^N\frac{\partial c_v}{\partial t} = H\frac{\partial}{\partial N}\left[\left(A_{m,0}(2\zeta\sqrt{\beta})^N D_v H\frac{\partial c_v}{\partial N}\right) - \left(Q_{m,0}(2\varepsilon\zeta\sqrt{\beta})^N c_v\right)\right] + \text{Source} - \text{Sink} \tag{S13}$$

The above equation is further reduced by multiplying and dividing by $\left(\frac{L_0}{A_{m,0}D_v}\right)$ and $\left(-\frac{\alpha\ \text{ln}(\alpha)}{1-\alpha}\right)$, respectively. The reduced equation is expressed as

$$\begin{aligned}\frac{L_0|V_{m,0}|}{D_v}(2\alpha\zeta\sqrt{\beta})^N T_m\frac{\partial c_v}{\partial t} = \frac{\partial}{\partial N}\left[\left(\left(\frac{2\zeta\sqrt{\beta}}{\alpha}\right)^N\left(\frac{1-\alpha}{\alpha\ \text{ln}(\alpha)}\right)^2\frac{\partial c_v}{\partial N}\right) - \left(\frac{L_0|V_{m,0}|}{D_v}(2\varepsilon\zeta\sqrt{\beta})^N c_v\right)\right] \\ + \left(\phi_l L'_D\frac{A_0 D_d}{L_0^2\alpha^N}c_d\frac{L_0^2\alpha^N}{A_{m,0}D_v}\right) + \left(A_m(pI - cc_v)\frac{L_0^2\alpha^N}{A_{m,0}D_v}\right)\end{aligned} \tag{S14}$$

The following parameters (Eqs. S15 and S16) are utilised to achieve the dimensionless form of the drug molecule transport equation in the airway mucus given by Eq. S17.

$$\begin{aligned}\tau = \frac{t}{T_b}, \phi_v = \frac{c_v}{c_{v,0}}, St_m = \frac{T_m}{T_b}, Pe_v = \frac{|V_{m,0}|L_0}{D_v}, p_0 = \frac{L_0^2}{D_v}\frac{p}{c_{v,0}}, \\ c_l = \frac{L_0^2}{D_v}c, I_r = \beta c_{v,0}T_b, \tau_E = \frac{T_E}{T_b}, \tau_I = \frac{T_I}{T_b}\end{aligned} \tag{S15}$$

$$c_{v,0} = \phi_l c_{d,0}\frac{A_0}{A_{m,0}}, T_m = \frac{L_0}{|V_{m,0}|}, D_v = \frac{k_B T}{3\pi\mu_m d_v} \tag{S16}$$

$$\begin{aligned}|Pe_v|(2\alpha\zeta\sqrt{\beta})^N St_m\frac{\partial\phi_v}{\partial\tau} = \frac{\partial}{\partial N}\left[\left(\left(\frac{2\zeta\sqrt{\beta}}{\alpha}\right)^N\left(\frac{1-\alpha}{\alpha\ \text{ln}(\alpha)}\right)^2\frac{\partial\phi_d}{\partial N}\right) - \left(|Pe_d|(2\varepsilon\zeta\sqrt{\beta})^N\phi_d\right)\right] \\ + \left(L'_D\frac{D_d}{D_v}\phi_d\right) + (2\alpha\zeta\sqrt{\beta})^N\left(p_0 I - c_l\phi_v\right)\end{aligned} \tag{S17}$$

where, ϕ_d, Pe_d and St_m represents the dimensionless drug concentration, Peclet number for the drug molecules and Strouhal number for the mucus layer, respectively. T_m denotes the time-scale for mucociliary transport. Eq. S17 is equivalent to Eq. 3 in the main manuscript.

The equations governing the kinetics of the virus infection are reduced to their dimensionless forms (Eqs. 4-6 in the main manuscript) as follows -

$$\begin{aligned} &\frac{\partial T}{\partial t} = -\beta T c_v \\ \implies &\frac{1}{T_b}\frac{\partial T}{\partial \tau} = -\beta T \phi_v c_{v,0} \\ \implies &\frac{\partial T}{\partial \tau} = -\beta c_{v,0} T_b T \phi_v \\ \implies &\frac{\partial T}{\partial \tau} = -I_r T \phi_v \end{aligned} \tag{S18}$$

$$\begin{aligned} &\frac{\partial E}{\partial t} = \beta T c_v - \frac{1}{T_E} E \\ \implies &\frac{1}{T_b}\frac{\partial E}{\partial \tau} = \beta T \phi_v c_{v,0} - \frac{1}{T_E} E \\ \implies &\frac{\partial E}{\partial \tau} = \beta c_{v,0} T_b T \phi_v - \frac{T_b}{T_E} E \\ \implies &\frac{\partial E}{\partial \tau} = I_r T \phi_v - \frac{1}{\tau_E} E \end{aligned} \tag{S19}$$

$$\begin{aligned} &\frac{\partial E}{\partial t} = \frac{1}{T_E} E - \frac{1}{T_I} I \\ \implies &\frac{1}{T_b}\frac{\partial I}{\partial \tau} = \frac{1}{T_E} E - \frac{1}{T_I} I \\ \implies &\frac{\partial I}{\partial \tau} = \frac{T_b}{T_E} E - \frac{T_b}{T_I} I \\ \implies &\frac{\partial I}{\partial \tau} = \frac{1}{\tau_E} E - \frac{1}{\tau_I} I \end{aligned} \tag{S20}$$

4 INFECTION KINETICS MODEL PARAMETERS FOR VALIDATION

Table S3 summarises the magnitudes of various model parameters used in the virus infection kinetics model for the comparative cases with SARS-CoV-2 and Influenza A (see Fig. 1 in the main manuscript). The immune model parameters are not listed for the Influenza A comparison since it was not considered in this case.

5 SUPPORTING RESULTS

5.1 Droplet deposition

Figure S2 compares the droplet deposition fraction, as determined using the developed mathematical model, with the experimental data of Heyder et al. (1986) for various droplet sizes. The results are shown for the complete LRT as well as the deep lung specifically. It can be seen that the predicted deposition fraction of droplets over the entire size range is close to that observed experimentally, indicating the adequacy of this model in determining droplet transport and deposition in the LRT including the deep lung.

Figure S3 shows the spatial change in droplet deposition along the LRT, at the end of droplet inhalation, for the baseline parameters (see Table 1 in the main manuscript). It is observed that the spatial distribution

Table S3. Dimensional magnitudes of infection kinetics parameters used in comparing the present model with SARS-COV-2 and Influenza-A viral load.

Model Parameter	SARS-CoV-2	Influenza-A
p	1.48×10^{11} virus.ml^{-1}d^{-1}	7.67×10^{5} virus.ml^{-1}h^{-1}
c	10 d^{-1}	0.22 h^{-1}
β	1.46×10^{-5} ml.virus^{-1}d^{-1}	1.33×10^{-6} ml.virus^{-1}h^{-1}
T_E	3.03 h	8 h
T_I	0.416 h	20 h
f	0.5	n/a
$t_{p,i}$	3 d	n/a
$\lambda_{g,i}$	2 d^{-1}	n/a
$\lambda_{d,i}$	1 d^{-1}	n/a
k_A	1 h^{-1}	n/a
Ab_0	0.002	n/a
$\lambda_{g,a}$	0.75 d^{-1}	n/a
k_C	0.03 h^{-1}	n/a
$t_{p,t}$	8 d	n/a
$\lambda_{g,t}$	2 d^{-1}	n/a
$\lambda_{d,t}$	0.1 d^{-1}	n/a

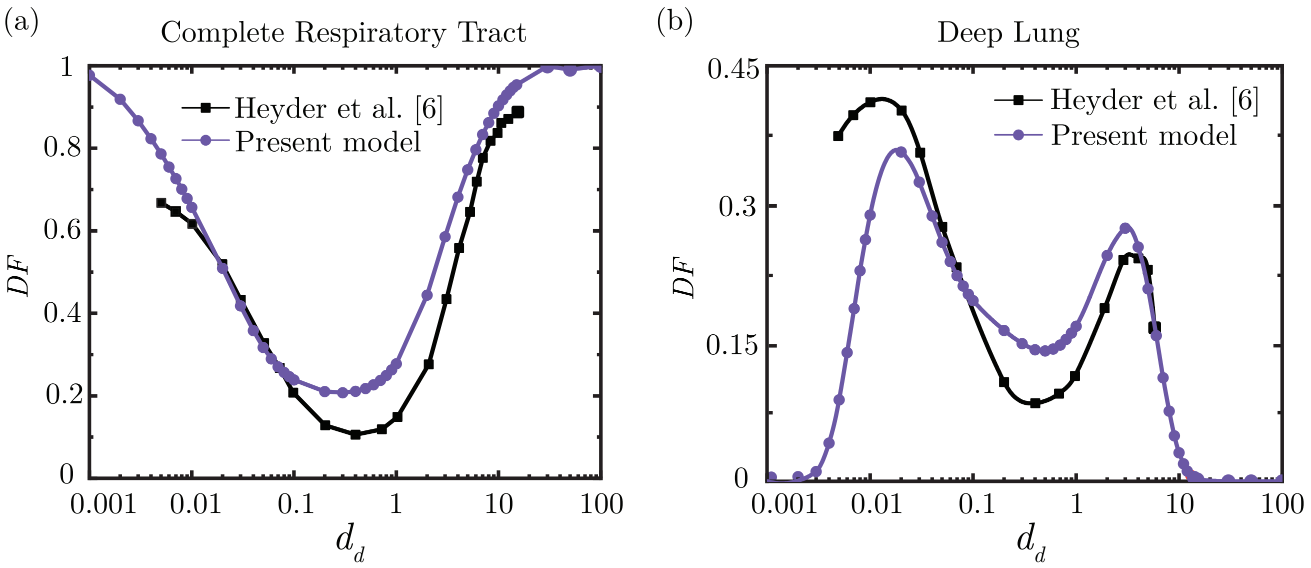

Figure S2. Comparison of calculated droplet deposition fraction (DF) with experimetal data of Heyder et al. Heyder et al. (1986) for different droplet sizes.

of virus concentration along the LRT, at the end of droplet inhalation (see Fig. 1a in the main manuscript), remains qualitatively similar to the droplet deposition characteristics.

5.2 Pneumonia time-estimate for SARS-CoV-2

REFERENCES

Chakravarty, A., Panchagnula, M. V., Mohan, A., and Patankar, N. A. (2022). Pulmonary drug delivery and retention: A computational study to identify plausible parameters based on a coupled airway-mucus flow model. *PLOS Computational Biology* 18, e1010143

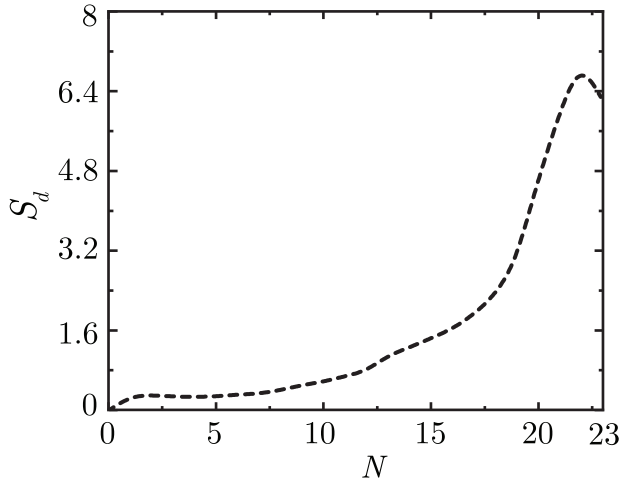

Figure S3. Deposition of virus-laden droplets ($S_d = \int\int L'_D \phi_d d\forall d\tau$) within the LRT at the end of droplet inhalation. The result is shown for the baseline parameters (see Table 1 in the main manuscript). $d\forall$ is the airway volume at each generation of the LRT.

Table S4. Variation in estimated time required for developing SARS-CoV-2 pneumonia with change in various fluid dynamic and physiological parameters, other parameters remaining constant. The results are shown considering different $c_{v,cr}$ in the deep lung.

**Parameter**		**Estimated time (days)**	
		$c_{v,cr} = 7 \times 10^6$	$c_{v,cr} = 5 \times 10^4$
d_d (μm)	100	5.65	4.26
	10	5.56	4.07
	5	5.23	3.75
	3	5.12	3.56
	2	5.14	3.66
	1	5.19	3.73
	0.5	5.23	3.79
d_v (μm)	0.01	5.25	3.8
	0.1	5.19	3.75
T_b (s)	1	6.02	4.63
	2	5.78	4.32
	4	5.19	3.73
	8	3.52	1.76
Q_0 (m^3/s)	7.088×10^{-4}	5.37	3.89
	7.875×10^{-4}	5.19	3.73
	8.66×10^{-4}	5.09	3.61
L_0 (m	0.108	5.09	3.61
	0.12	5.19	3.73
	0.132	5.32	3.84

Chakravarty, A., Patankar, N. A., and Panchagnula, M. V. (2019). Aerosol transport in a breathing alveolus. *Physics of Fluids* 31, 121901

Darquenne, C. and Paiva, M. (1994). One-dimensional simulation of aerosol transport and deposition in the human lung. *Journal of applied physiology* 77, 2889–2898

Devi, S. G. K. (2018). *Aerosol Deposition Studies in Human Lung - Towards Personalized Medicine*. Ph.D. thesis, IIT Madras, India

Devi, S. K., Panchagnula, M. V., and Alladi, M. (2016). Designing aerosol size distribution to minimize inter-subject variability of alveolar deposition. *Journal of Aerosol Science* 101, 144–155

Table S5. Variation in estimated time required for developing SARS-CoV-2 pneumonia with change in various infection parameters, other parameters remaining constant. The results are shown considering different $c_{v,cr}$ in the deep lung.

**Parameter**		**Estimated time (days)** $c_{v,cr} = 7 \times 10^6$	$c_{v,cr} = 5 \times 10^4$
p	5×10^{10}	No pneumonia onset	
	7×10^{10}	No pneumonia onset	6.16
	1.0×10^{11}	6.95	4.86
	1.1×10^{11}	6.25	4.54
	1.2×10^{11}	5.88	4.31
	1.3×10^{11}	5.61	4.07
	1.4×10^{11}	5.42	3.89
	1.48×10^{11}	5.23	3.75
c	0	2.55	1.71
	5	3.98	2.5
	10	5.23	3.75
	20	No pneumonia onset	5.6
	50	No pneumonia onset	
f	0	3.89	2.78
	0.2	No pneumonia onset	4.73
	0.4	5.42	3.94
	0.6	4.95	3.56
	0.8	4.72	3.38
	1	4.63	3.24
$t_{p,i}$	1	5.19	4.73
	3	5.19	3.7
	5	4.03	2.78
	7	3.8	2.69
	9	3.8	2.64
k_A	0	4.77	3.1
	0.5	4.86	3.19
	1	4.95	3.24
	5	5.7	3.38
	10	No pneumonia onset	3.84
A_0	0.002	5.19	3.73
	0.005	5.46	3.89
	0.01	5.64	3.98
	0.05	No pneumonia onset	4.81
	0.1	No pneumonia onset	5.42

Heyder, J., Gebhart, J., Rudolf, G., Schiller, C. F., and Stahlhofen, W. (1986). Deposition of particles in the human respiratory tract in the size range 0.005-15 μm. *Journal of aerosol science* 17, 811–825

Karamaoun, C., Sobac, B., Mauroy, B., Van Muylem, A., and Haut, B. (2018). New insights into the mechanisms controlling the bronchial mucus balance. *PloS one* 13, e0199319

Mauroy, B., Fausser, C., Pelca, D., Merckx, J., and Flaud, P. (2011). Toward the modeling of mucus draining from the human lung: role of the geometry of the airway tree. *Physical biology* 8, 056006

Mitsakou, C., Helmis, C., and Housiadas, C. (2005). Eulerian modelling of lung deposition with sectional representation of aerosol dynamics. *Journal of Aerosol Science* 36, 75–94

Taulbee, D. B. and Yu, C. (1975). A theory of aerosol deposition in the human respiratory tract. *Journal of Applied Physiology* 38, 77–85

Weibel, E. R., Cournand, A. F., and Richards, D. W. (1963). *Morphometry of the human lung*, vol. 1 (Springer)
